# *Haloxylon ammodendron* adapts to desert environments through seed polymorphism during diaspore germination and seedling establishment

**DOI:** 10.3389/fpls.2025.1527718

**Published:** 2025-04-10

**Authors:** Ziyi Wang, Weizhi Chen, Minghao Yang, Lamei Jiang, Ze Wang, Cai Ren, Xianhua Zhang

**Affiliations:** ^1^ College of Grassland Science, Xinjiang Agricultural University, Urumqi, China; ^2^ College of Life Sciences, Xinjiang Agricultural University, Urumqi, China; ^3^ Xinjiang Key Laboratory for Ecological Adaptation and Evolution of Extreme Environment Biology, College of Life Sciences, Xinjiang Agricultural University, Urumqi, China; ^4^ College of Resources and Environment, Xinjiang Agricultural University, Urumqi, China

**Keywords:** seed polymorphism, extreme environment, bionomic strategy, germination and seedling establishment, *Haloxylon ammodendron*

## Abstract

**Introduction:**

Seed polymorphism, defined as the production of two or more types of diaspores with distinct morphology and ecological function within a species, represents a bet-hedging strategy that enables plants to cope with unpredictable spatiotemporal environmental variability. Previous studies have mainly focused on annual plants; therefore, little is known about in perennial species, particularly in desert constructive plants.

**Methods:**

This study investigated seed polymorphism in *Haloxylon ammodendron*, a foundational desert shrub critical for maintaining the stability of fragile arid ecosystems. Field surveys, morphological characterization, phytohormone quantification, germination assays, and seedling growth analyses were conducted to elucidate the ecological significance of seed polymorphism in this species.

**Results and discussion:**

Seed polymorphism was prevalent across natural populations within the study region, with different plants producing three distinctly colored diaspores: YY (yellow fruit-wing perianth and yellow pericarp), YP (yellow fruit-wing perianth and pink pericarp), and PP (pink fruit-wing perianth and pink pericarp). The fruit/diaspore biomass and gibberellic acid/abscisic acid ratio were the lowest in YY (0.611 and 0.64, respectively) and the highest in YP (0.684 and 1.56). YY plants exhibited grater drought resistant and produced fewer but more robust seedlings, ensuring population persistence. YP seeds have a higher germination percentage, germination rate, and emergence percentage, facilitating rapid population expansion under favorable conditions. PP seeds showed reduced germination under salt stress, suggesting a potential role as a persistent soil seed bank. These results indicate that *H. ammodendron* employs seed polymorphism to adapt to unpredictable desert environment during diaspore germination and seedling establishment. This study enhances the theoretical understanding of the bionomic strategies underpinning plant adaptation to extreme environments, with implications for population persistence and regeneration dynamics, while also providing diversified germplasm resources for desertification prevention.

## 1 Introduction

Charles Darwin famously stated: “It is not the strongest of the species that survives, nor the most intelligent that survives. It is the one that is the most adaptable to change.” To survive in highly unpredictable spatiotemporally changing environments such as deserts, saltmarshes, and gravel, plants had to evolve unique bionomic strategies. One of the strategies, seed polymorphism, has important value in research on plant ecological adaptation strategies and evolutionary mechanisms (Harper et al., 1970; Imbert, 2002; Orlovsky et al., 2016; Hasanuzzaman et al., 2019). Generally, for individual plant or population, the size and shape of the seeds they produce exhibit a continuous variation. However, some species characteristically produce two or more sharply defined types of seed that often exhibit significant differences in morphological features (e.g., color, size, and internal structure) and ecological behaviors (e.g., dispersal, germination, and seedling growth), which is defined as seed polymorphism (Harper et al., 1970). Seed polymorphism can take various forms: (1) genetic polymorphism, which refers to the occurrence of two or more forms of a species in the same habitat in such proportions that the rarest of them cannot be maintained by recurrent mutation and (2) seed heteromorphism, which refers to the production of two or more diaspores by individual plants (Harper et al., 1970; Wang et al., 2010; Fenesi et al., 2019; Baskin and Baskin, 2024).

Polymorphic seeds with distinct external morphologies often exhibit differences in dispersal, germination, and stress responses (Takeno and Yamaguchi, 1991; Whitney and Lister, 2004; Yang et al., 2015; Čalasan and Kadereit, 2023). Seed polymorphism occurs in more than 378 plants in 129 genera of 26 families, mainly in Asteraceae, Amaranthaceae, Leguminosae, Brassicaceae, and Caryophyllaceae. Most of these are annuals, with only slightly more than 20 perennials (Wang et al., 2008; Scholl et al., 2020). Our survey area, the Gurbantunggut Desert located in the Junggar Basin, northern Xinjiang, China, harbors more than 20 plant species with seed polymorphism (Baskin et al., 2014).

**Figure 1:**
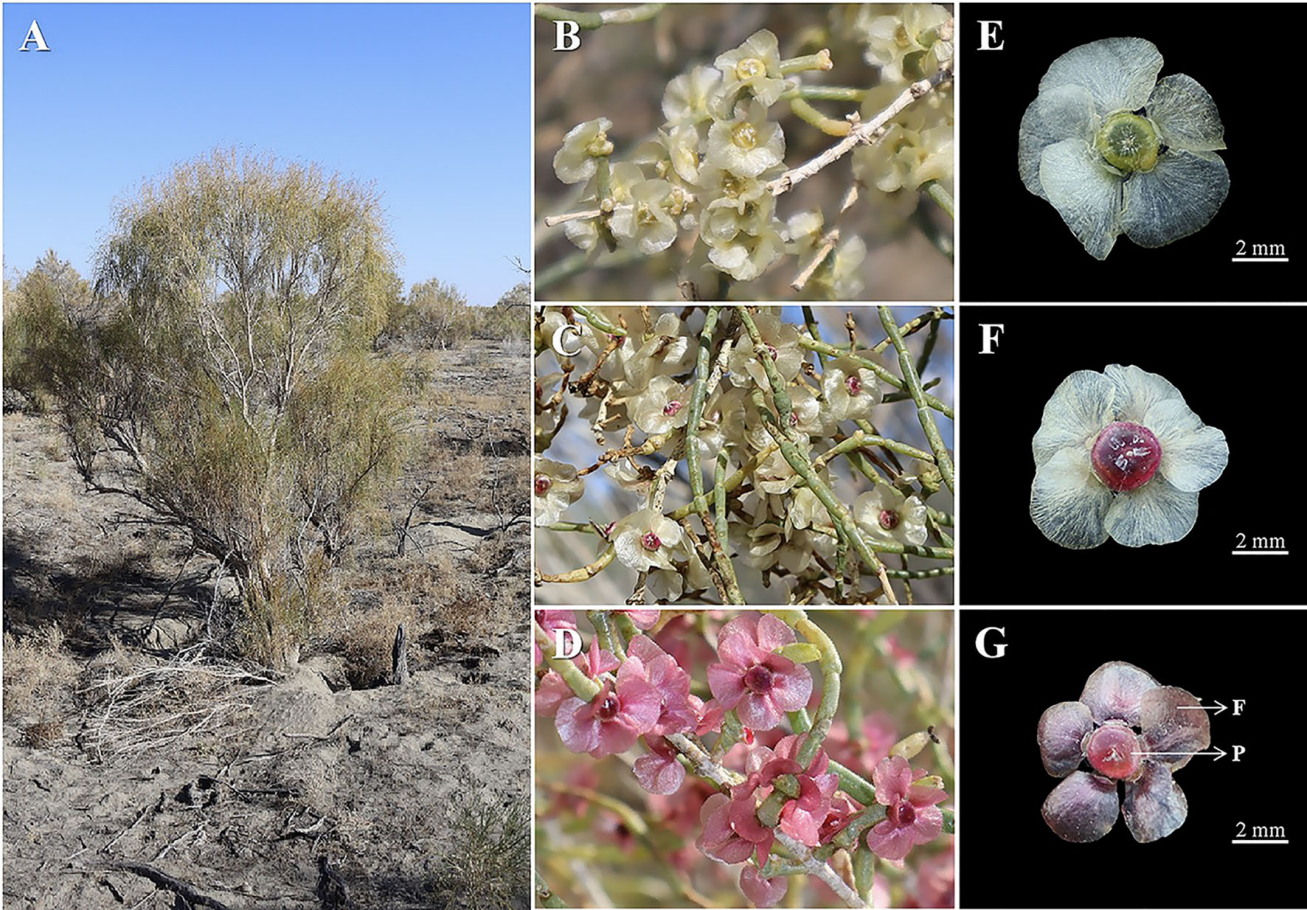
Habitat of *Haloxylon ammodendron* **(A)** and the three types of plants with differently colored diaspores: YY, with yellow fruit-wing perianth and yellow pericarp **(B, E)**, YP, with yellow fruit-wing perianth and pink pericarp **(C, F)**, and PP, with pink fruit-wing perianth and pink pericarp **(D, G)**. Diaspores are characterized by five fruit-wing perianths (marked as “F”) and one pericarp (marked as “P”) **(G)**.

*Haloxylon* (Amaranthaceae) is a genus of Mediterranean relic shrubs widely distributed in the deserts of the Eurasian continent that play an invaluable role in maintaining ecosystem stability and preventing desertification in this region. The genus comprises approximately 11 species. In China, there are only two species, *H. ammodendron* and *Haloxylon persicum*. *H. ammodendron* is mainly distributed in Xinjiang, Gansu, Qinghai, Ningxia, and Inner Mongolia (Pyankov et al., 1999; Yang et al., 2014). *H. ammodendron* is mainly distributed in the Gurbantunggut Desert of the Junggar Basin in Xinjiang, where it serves as an important constructive species. The community with *H. ammodendron* as the dominant species provides shelter for nearly 200 species (Sheng et al., 2005). In the natural environment of this area, *H. ammodendron* diaspores germinate from March to April, flowers are hermaphrodite, and the pollination mode is the main mode of facultative outcrossing. The diaspores are composed of one ventricle and five fruit-wing perianths that appear during the fruit ripening period from September to October (Chen and Zuo, 2019; Zhou and Gong, 2019). In this study, we found *H. ammodendron* plants with three differentially colored diaspores within the same population distributed in the Gurbantunggut Desert: yellow fruit-wing perianth and yellow pericarp (YY), yellow fruit-wing perianth and pink pericarp (YP), and pink fruit-wing perianth and pink pericarp (PP) (**Figure 1**). According to our observations over the past 5 years had demonstrated that diaspores of the same plant had a single-color pattern. Based on the above findings, we propose that *H. ammodendron* exhibits seed polymorphism (genetic polymorphism).

In the present study, we aimed to explore whether the seed polymorphism of *H. ammodendron* is widespread in natural habitats and whether differences exist in the morphological characteristics and ecological behavior of diaspores and the ecological significance thereof. To this end, we investigated *H. ammodendron* plants in multiple sample plots in the natural distribution area of *H. ammodendron* in the Gurbantunggut Desert and compared the biomass, endogenous hormone levels, and germination and seedling growth characteristics among plants with different types of diaspores. We expected our study to provide a theoretical basis for revealing the mechanisms of plant adaptation to the unpredictable desert environment.

## 2 Materials and methods

### 2.1 Study population and sampling

The environmental conditions of the Junggar Basin are complex and variable. Winter is cold, with an average temperature of -17°C in December and a minimum temperature of less than -30°C, whereas summer is hot, with an average temperature of 27°C in July and a maximum temperature of more than 45°C, and the diurnal temperature range is large (often > 10°C) (Li et al., 2014). The area is also one of the main distribution areas of soil salinization in China, with up to 10 g salt/kg soil. Precipitation is scarce, with an average annual value of 70-150 mm. And precipitation is mainly concentrated in winter and spring, which account for 30%-45% of the annual precipitation (Peng et al., 2017; Ding and Peng, 2020). Moreover, the crisscross sand dunes cause a high degree of spatiotemporal heterogeneity in temperature, salinity, and water conditions. This highly unpredictable environment is the main ecological factor restricting the population persistence of *H. ammodendron*.

**Figure 2:**
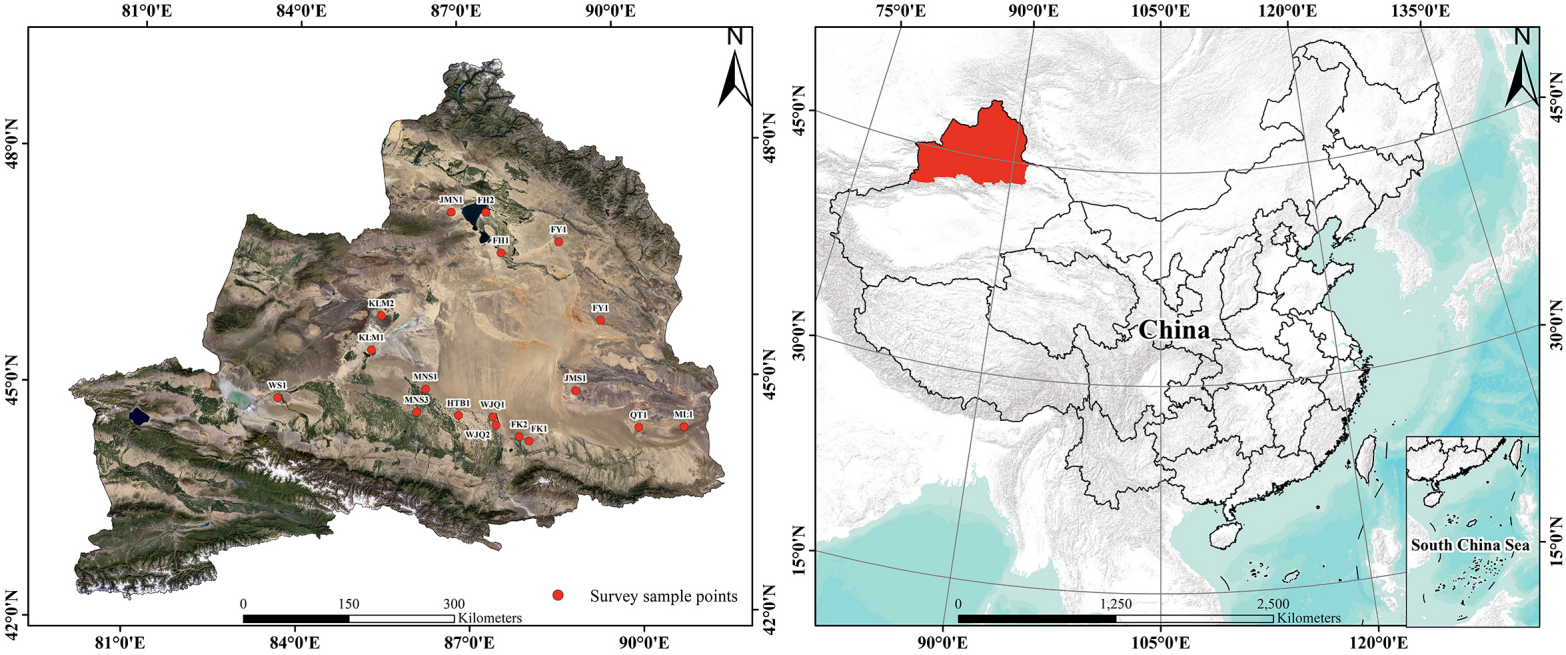
Distribution of the 18 sampling plots of *Haloxylon ammodendron* in the Gurbantunggut Desert of the Junggar Basin, Xinjiang, China. In the figure, abbreviations represent geographic names: FH, Fuhai; FK, Fukang; FY, Fuyun; WS, Wusu; HTB, Hutubi; JMS, Jimusaer; KLM, Karamay; ML, Mulei; SHZ, Shihezi; QT, Qitai; WJQ, Wujiaqu.

We conducted a field survey in the natural distribution area of *H. ammodendron* in the Gurbantunggut Desert in October 2023, which was the fruit ripening period, and the color differences of diaspores were clearly visible. A total of 18 sample plots were set up in the survey area, and GPS (DCM-104, Dong Mei Measuring Instrument Co., Ltd, Shenzhen, China) was used to record the latitude, longitude, and altitude. Each plot had an area of 100 m × 100 m (**Figure 2**). The raw data of sampling points (latitude and longitude) were processed using Excel 2016 (Microsoft, Redmond, Washington, USA, 2016), and subsequently imported into ArcGIS 10.2 (ESRI, Redlands, California, USA, 2013) for **Figure 2** generation. Plants with three differently colored diaspores were distinguished, labeled, and counted separately in each plot.

**Figure 3:**
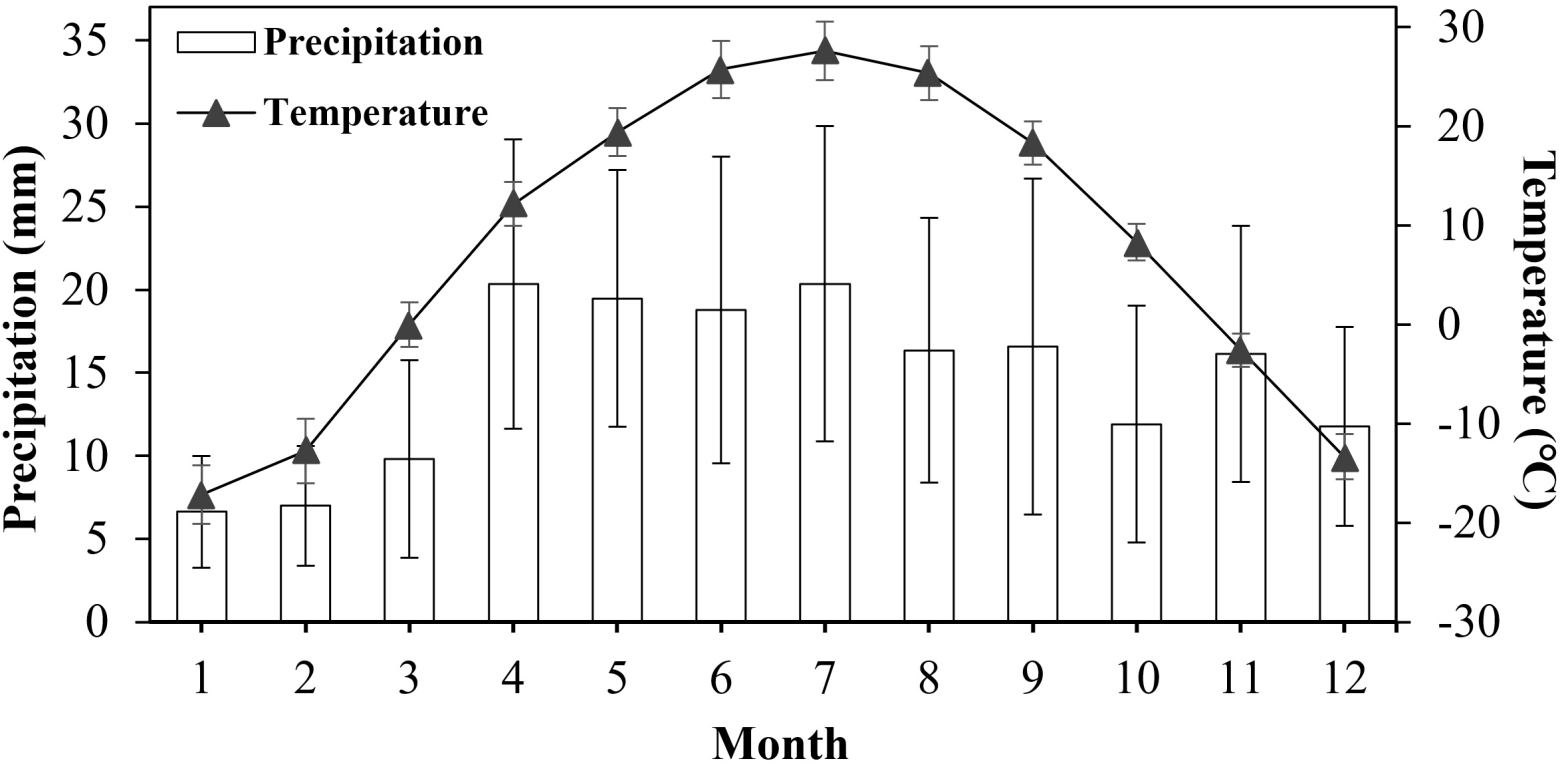
Average monthly precipitation and temperature at the Fukang, China sampling site from 2001 to 2022. Data are means ± standard deviations.

Fukang (44.37°N, 87.96°E), China. with well-growing plants, was selected as the diaspore collection site. It is characterized by a typical temperate continental arid and semi-arid climate, and the annual average precipitation is 145.9 mm. The annual average temperature is 7.6°C, the highest monthly average temperature is 28.6°C in July, and the lowest is -17.1°C in January (**Figure 3**). In November 2023, when the diaspores had become dehydrated and ripened, complete diaspores of the three types were collected separately into boxes and dried naturally in a dark laboratory at room temperature.

### 2.2 Analysis of weight and determination of phytohormone concentrations

To evaluate variations in weight and phytohormone concentration across three types of diaspore, a single-factor experimental design was implemented. The dispersal units were air-dried in dark, impurities were removed, and they were stored in a refrigerator at 4°C for subsequent use. (1) Analysis of weight: The diaspores were weighed using 0.001-g electronic balance in four replicates, with 1,000 diaspores per replicate. After the fruit-wing perianth of each diaspore was removed, the fruits were weighed again, and the weight proportion of each part calculated. (2) Determination of phytohormone concentrations: Endogenous gibberellic acid (GA) and abscisic acid (ABA) concentrations are related to seed dormancy and germination ability. So, the diaspores were weighed in four replicates, thoroughly ground in liquid nitrogen, and then mixed with the extraction buffer (0.1mmol PH 7.4 PBS). After extraction at 4°C for 2 hours, the mixture was centrifuged for 10 minutes, and the supernatant was collected. Thus, phytohormone concentrations were determined by the plant GA/ABA enzyme-linked immunosorbent assay ELISA kit using Shanghai Xuanzecang kit and microplate reader (SpectraMax Mini, Molecular Devices Co., Ltd., Shanghai. China).

### 2.3 Analysis of germination characteristics under different temperature conditions

To explore the adaptive strategies and ecological significance of seed polymorphism in *H. ammodendron*, we used a single-factor experimental design to assess its responses to temperature, salinity and moisture, respectively. To explore the adaptive strategies and ecological significance of seed polymorphism in *H. ammodendron*, we assessed its responses to temperature, salinity, and moisture. Diaspores were placed in a 100 mm petri dish (Labselect, Anhui, China) containing two 9cm-diameter filter papers and 10 mL of distilled water and incubated in illuminated incubators (GXZ-280B, Ningbo Jiangnan Instrument Factoy, Zhejiang, China) at 2°C/5°C, 5°C/15°C, and 15°C/25°C (12 h dark/12 h light), corresponding to the average temperatures in the Fukang sample plot in March, April, and May, respectively (**Figure 3**).Four replicates were set for each temperature treatment group, with 25 diaspores per replicate. The germination status was monitored—germination was defined as embryonic root protrusion—and germinated seeds were counted and pickde out. An appropriate amount of distilled water was added every day to keep the filter paper moist. The experiment lasted 14 days. The germination percentage (GP) and germination rate index (GRI) were calculated using the following formulae:


GP=N/S*100%

GRI=∑(n/D)/S

where N is the number of germinated seeds, S is the number of diaspores tested, *n* is the number of germinated seeds on day D, and D is the number of days required for germination (the maximum value of GRI is 100) (Hsu et al., 1985).

### 2.4 Analysis of germination characteristics under different salt conditions

To evaluate salt tolerance in diaspore germination, a single-factor design was utilized, comparing germination rates across diaspore types under controlled saline conditions at the same level. Diaspores were germinated as mentioned above in the presence of NaCl at 0, 0.1, 0.2, 0.4, 0.6, and 0.8 mol/L in an incubator at 5°C/15°C (12 h dark/12 h light) (Zhumabekova et al., 2020). Four replicates were set for each NaCl treatment group, with 25 diaspores per replicate. The petri dishes were weighed daily, and an appropriate amount of distilled water was added to maintain a constant salt concentration for 28 days. The germination status was monitored and recorded as described above.

### 2.5 Analysis of seedling growth under different salt and water conditions

A single-factor experimental design was employed to compare seedling-growth characteristics among three types of diaspores under different salt and water conditions. Thirty diaspores of each type were seeded in a pot (30-cm diameter and depth) filled with desert native soil, with 6 pots per treatment. The pots were placed in a greenhouse from 18 May to 14 June 2023 (30 ± 5°C). Soil NaCl contents of 0, 7, 13, and 16 g/kg were tested based on the desert native soil conditions (Agricultural Bureau and Soil Survey Office of Uygur Autonomous region of Xinjiang, 1996), and the pots were watered to field capacity every day (soil water content, 20%). In addition, three watering regimes were tested, with water being added to field capacity every day, every 3 days, or every 6 days. Emerging seedlings (defined as cotyledon emergence from the soil) were counted once daily for 28 days, and the seedling survival percentage (SP) was determined. The seedlings were collected and cleaned without damaging the root system and transferred to the laboratory for biomass measurement using a 0.0001-g electronic balance. The emergence percentage (EP) and SP were calculated as follows:


EP=N/S*100%

SP=n/N*100%

where N is the number of emerged seedling, S is the number of diaspores tested, and *n* is the number of surviving seedlings on day 32.

### 2.6 Statistical analysis

The Shapiro–Wilk test was used to test normal distribution, and Levene’s test was performed to confirm homogeneity of variance (*p* > 0.05). When the data followed a normal distribution with homogeneity of variance, one-way ANOVA and the Student–Neumann–Keuls test were used. When the data did not meet the requirement of homogeneity of variance, the non-parametric Tamhane’s T2 test was used. Statistical significance was set to *p* < 0.05. All statistical analyses were performed using SPSS 25 (SPSS, Los Angeles, CA, USA).

## 3 Results

### 3.1 Seed polymorphism of *H. ammodendron* is common in the survey area

We investigated the natural distribution area of *H. ammodendron*, which is widespread in the Gurbantunggut Desert of the Junggar Basin. Each of the 18 sampling plots had plants with the three types of differently colored diaspores, and each individual plant had only one type of diaspores (**Figure 2**, **Supplementary Table S1**). Diaspores are composed of a fruit-wing perianth and pericarp, and their colors could be clearly distinguished during the ripening period in October.

### 3.2 Morphological characteristics and phytohormone concentrations

We observed significant differences in morphological and physiological characteristics among the different types of diaspores (**Table 1**). Regarding the morphological characteristics, besides color, biomass differences were reflected in the weight of each structure and the weight proportion. The biomass of YP perianth was medium (1.008 ± 0.022 g), whereas the biomass of YP fruits and diaspores and the fruit/diaspore weight ratio were higher than those of the other types. YY had the lowest fruit/diaspore weight ratio (0.611 ± 0.016). Regarding phytohormone concentrations, YP diaspores had the lowest ABA concentration (215.42 ± 1.17 ng/g), whereas its GA concentration and GA/ABA concentration ratio were significantly higher than those of the other types, and YY had the lowest GA/ABA concentration ratio (0.64 ± 0.04) (**Table 1**).

**Table 1:** Comparative morphological and biophysical properties of polymorphic diaspores of *Haloxylon ammodendron*.

Attributes	YY	YP	PP
Fruit (g)	1.937 ± 0.059 b	2.179 ± 0.035 a	1.765 ± 0.023 c
Perianth (g)	1.234 ± 0.048 a	1.008 ± 0.022 b	0.938 ± 0.013 c
Diaspore (g)	3.171 ± 0.012 a	3.187 ± 0.014 a	2.702 ± 0.023 b
Fruit/diaspore weight ratio	0.611 ± 0.016 c	0.684 ± 0.008 a	0.653 ± 0.005 b
GAs (ng/g)	289.60 ± 2.12 c	334.49 ± 5.25 a	318.60 ± 3.26 b
ABA(ng/g)	457.46 ± 35.16 a	215.42 ± 1.17 c	371.38 ± 9.71 b
GA/ABA content ratio	0.64 ± 0.04 c	1.56 ± 0.02 a	0.86 ± 0.01 b

Different letters in each row indicate significant differences at *P* < 0.05. Data are means ± standard deviations.

### 3.3 Germination characteristics under different temperature conditions

The *indoor germination experiment* germination experiment revealed no significant differences in the GP of the three types of diaspores under different temperature conditions, but the GRI did differ (**Table 2**). Under all three temperature conditions, the GRI of YP was higher than that of the other types, particularly at 2°C/5°C, whereas that of YY was the lowest (**Table 2**).

**Table 2:** Comparative germination characteristics of polymorphic diaspores of *Haloxylon ammodendron* at different temperatures.

Attributes	Temperature (°C) (dark/light)			
		YY	YP	PP
GP (%)	2/5	89.0 ± 6.8 a	91.0 ± 8.9 a	93.0 ± 3.8 a
	5/15	100.0 ± 0.0 a	100.0 ± 0.0 a	100.0 ± 0.0 a
	15/25	100.0 ± 0.0 a	100.0 ± 0.0 a	100.0 ± 0.0 a
GRI	2/5	23.1 ± 3.8 b	34.3 ± 1.2 a	27.5 ± 2.8 b
	5/15	37.5 ± 4.0 b	47.8 ± 3.4 a	46.9 ± 3.1 a
	15/25	49.7 ± 5.6 b	68.7 ± 6.0 a	67.4 ± 3.7 a

Different letters in each row indicate significant differences at *P* < 0.05. Data are means ± standard deviations.

GP, germination percentage; GRI, germination rate index.

### 3.4 Germination characteristics under different salt conditions

**Figure 4:**
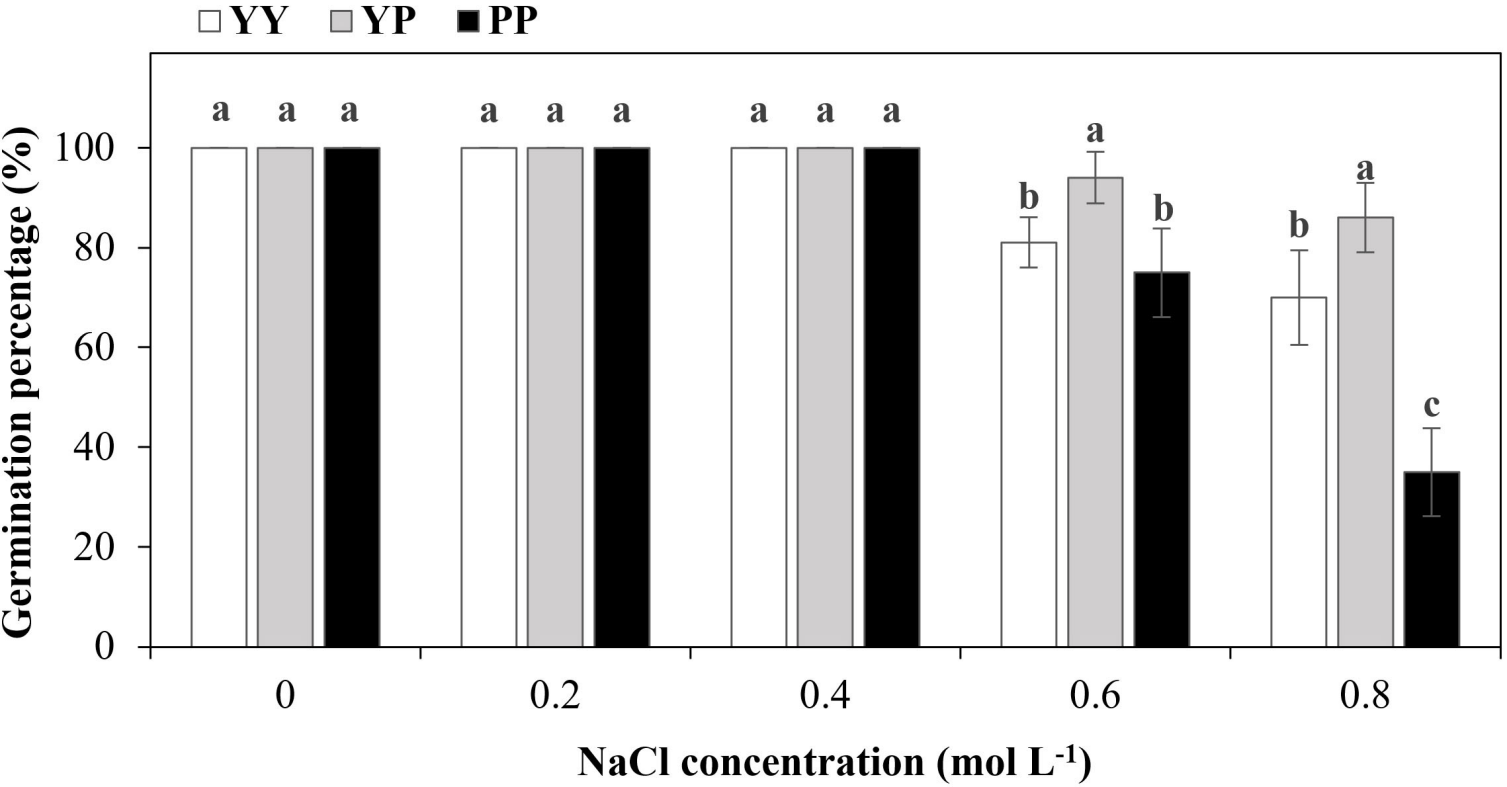
Final germination percentage of polymorphic diaspores in *Haloxylon ammodendron* under different NaCl concentrations. Different letters above bars indicate significant differences at *P* < 0.05. Data are means ± standard deviations.

The GPs were all 100% at NaCl ≤ 0.4 mol/L. With increasing NaCl concentration (0.6 and 0.8 mol/L), the GP of YP was remarkably higher than that of the other types (94.0% and 86.0%, respectively), whereas that of PP was the lowest (75.0% and 35.0%) (**Figure 4**). When the NaCl concentration was 0.8 mol/L, significant differences in germination rates were observed among the three types of dispersal units, with the YP type exhibiting the highest germination rate and the PP type showing the lowest (**Figure 4**).

### 3.5 Seedling growth under different salt and water conditions

Under greenhouse conditions, the EP, SP, and biomass of the polymorphic diaspores differed significantly under the various salt and water treatments (**Table 3**). In all salt treatments, the EP of YP was higher than that of the other types. The SP of the polymorphic diaspores were no significant difference at low NaCl concentrations (0 and 7 g/kg); however, YP had the lowest SP at a high NaCl concentration (13 g/kg), and PP consistently had the lowest biomass. In all water treatments, the EP of YP was higher than that of the other types, whereas the SP of the polymorphic diaspores did not differ. YP had higher biomass than the other types under daily watering, whereas YY had the highest biomass with 3- or 7-day watering intervals. These findings indicated that YY plants grow better than plants with other diaspore types under moderate drought conditions (**Table 3**).

**Table 3:** Comparative seedling growth characteristics of polymorphic diaspores of *Haloxylon ammodendron* under different NaCl concentrations and watering conditions.

Treatments	Attributes	Levels	YY	YP	PP
NaCl concentration (g/kg)	EP (%)	0 (g/kg)	22.2 ± 9.6 c	54.2 ± 9.6 a	36.7 ± 3.9 b
		7 (g/kg)	18.7 ± 3.8 c	50.0 ± 9.8 a	34.7 ± 8.0 b
		13 (g/kg)	21.7 ± 9.6 b	42.7 ± 5.5 a	18.7 ± 6.5 b
		16 (g/kg)	20.6 ± 9.1 b	40.8 ± 6.9 a	12.7 ± 6.0 b
	SP (%)	0 (g/kg)	91.0 ± 10.8 a	91.8 ± 5.9 a	88.3 ± 12.8 a
		7 (g/kg)	74.5 ± 11.2 a	69.3 ± 16.0 a	63.9 ± 14.5 a
		13 (g/kg)	49.0 ± 18.7 a	18.3 ± 10.7 b	38.9 ± 14.6 ab
		16 (g/kg)	–	–	–
	DW (mg)	0 (g/kg)	6.55 ± 0.25 b	7.46 ± 0.32 a	5.20 ± 0.20 c
		7 (g/kg)	4.76 ± 0.81 a	5.07 ± 0.50 a	3.84 ± 0.96 a
		13 (g/kg)	2.78 ± 0.15 a	2.67 ± 0.34 a	1.31 ± 0.21 b
		16 (g/kg)	–	–	–
Watering frequency	EP (%)	1d	22.2 ± 9.6 c	54.2 ± 9.6 a	36.7 ± 3.9 b
		3d	15.8 ± 3.2 c	41.7 ± 6.4 a	23.9 ± 4.9 b
		6d	14.7 ± 3.8 b	36.7 ± 8.6 a	12.0 ± 9.9 b
	SP (%)	1d	91.0 ± 10.8 a	91.8 ± 5.9 a	88.3 ± 12.8 a
		3d	84.6 ± 10.8 a	81.5 ± 9.3 a	85.1 ± 11.7 a
		6d	84.3 ± 15.1 a	79.8 ± 21.2 a	91.0 ± 21.2 a
	DW (mg)	1d	6.55 ± 0.25 b	7.46 ± 0.32 a	5.20 ± 0.20 c
		3d	8.41 ± 0.21 a	6.67 ± 0.29 b	6.73 ± 0.43 b
		6d	13.03 ± 0.51 a	7.08 ± 0.19 b	5.84 ± 0.26 c

Different letters in each row indicate signiﬁcant differences at *P* < 0.05. Data are means ± standard deviations.

EP, emergence percentage; SP, survival percentage; DW, dry weight; -: no data (seedlings were severely damaged due to the salt treatment).

## 4 Discussion

### 4.1 Seed polymorphism of *H. ammodendron* is common in the survey area

*H. ammodendron* is mainly distributed in the periphery of the Junggar Basin, whereas *H. persicum* occupies the middle area. We found all three types of plants in each survey plot, indicating that seed polymorphism of *H. ammodendron* is widespread in the study area. A previous study revealed that seed polymorphism also occurs in *Haloxylon salicornicum* distributed in Doha, Qatar (Bhatt et al., 2017a). The researchers found two types of diaspores, i.e., with yellow and pink fruit-wing perianths, which were distributed in the same population and exhibited significant differences in morphological characteristics and ecological behavior.

### 4.2 Morphological characteristics

Baskin and Baskin (2024) reported that seed polymorphism is first represented in morphological characteristics such as color, size, and position on the parent plant. *H. ammodendron* diaspores are composed of a fruit-wing perianth and a fruit. During the flowering period, the fruit wing is a persistent perianth hidden in the bracts and therefore, difficult to observe. The fruit wing, which assists in fruit spread, extends from the bracts during fruit ripening in autumn (Zhou and Gong, 2019). Based on the color of the fruit wing and pericarp, diaspores could be divided into three types, including YY, with a yellow fruit-wing perianth and yellow pericarp, YP, with a yellow fruit-wing perianth and pink pericarp, and PP, with a pink fruit-wing perianth and pink pericarp, whereas individual plants consistently produced only one type of diaspore. Based on these findings, we suggest that the seed polymorphism of *H. ammodendron* is due to genetic polymorphism (Harper et al., 1970; Baskin and Baskin, 2024).

Besides color, this research also found differences in the biomass and weight ratios of the fruit, fruit wing, and diaspore: YY had the lowest fruit/diaspore biomass ratio. YP had the highest fruit and diaspore biomass and fruit/diaspore biomass ratio, whereas PP had the lowest fruit and diaspore biomass. This is consistent with the morphological characteristics of seed polymorphism in *Atriplex canescens* and *H. salicornicum* (Amaranthaceae), which exhibit significant differences in diaspore color and biomass (Bhatt and Santo, 2016; Bhatt et al., 2017a). The weight of brown seeds in *A. canescuns* are significantly higher than that of black seeds, and in *H. salicornicum*, yellow diaspores are heavier than pink diaspores.

Endogenous hormone levels also differed among the polymorphic diaspores. The GA/ABA content ratio was the lowest in YY seeds and the highest in YP seeds. Seed size and biomass are often positively correlated with germination characteristics. For example, in *Salsola vermiculata*, the germination rate of yellow fruit-wing diaspores with higher biomass is significantly higher than that of pink counterparts with lower biomass (Bhatt et al., 2017b). Evidence suggests that the seed GA/ABA ratio is correlated with dormancy and germination, with a higher GA/ABA ratio indicating stronger germination potential (Khan, 1975; Bassel, 2016). Based on these findings, YP diaspores might have a better germination capacity because of their higher ratios of fruit/diaspore biomass and GA/ABA contents.

### 4.3 Ecological function of seed polymorphism

The polymorphic diaspores could germinate at 2°C/5°C, 5°C/15°C, and 15°C/25°C (dark/light), and significant differences were observed in the GRI rather than the GP. The GRI of YP was higher than that of the other types, particularly at 2°C/5°C, whereas that of YY was the lowest. This difference may be related to differences in diaspore biomass, hormones, and germination ability. Studies have shown that larger seeds germinate faster, and a high GA/ABA content ratio promotes germination (Carter and Ungar, 2003; Xian et al., 2024). Because of its higher fruit biomass, fruit/diaspore biomass ratio, and GA/ABA content ratio, YP had a high GRI, whereas YY had the lowest. *H. ammodendron* can germinate at low temperatures under snow cover in spring (Liu et al., 2019). The advantage of asynchronous germination of the polymorphic diaspores can be described as follows: in natural conditions, YP seeds germinate earlier and faster by fully utilizing the melted snow water to increase the survival probability, whereas seeds of the other two types germinate slowly and can be considered to form a short-lived soil seed bank that germinates later, in more appropriate circumstances, thus avoiding intraspecific competition for limited resources.

At relatively low salt concentrations (≤0.4 mol NaCl/L), the *indoor germination experiment* GPs of the different types of diaspores were all 100%. With increasing NaCl concentration (0.6 and 0.8 mol/L), the GP of YP was significantly higher than that of the other types, whereas that of PP was the lowest. This discrepancy might be because of the higher biomass of YP fruits. Compared with small seeds, large seeds can generate/accumulate more organic materials that act in reactive oxygen species detoxification and as signaling compounds to trigger responses to alleviate salt stress, thus improving the GP of seeds under salt stress (Liu et al., 2018).

Under natural conditions, the stress imposed by salinity and moisture is crucial for seedling establishment. In general, the germination rate shows a significant negative correlation with salt concentration. For example, seed germination rate of *H. ammodendron* decreases with increasing salt concentration (Huang et al., 2003). The response to drought stress varies significantly among different plant species. *Zygophyllum xanthoxylum* (Zygophyllaceae)seed germination rate significantly decreases under water potential conditions below -0.3 MPa, and can’t germination when below -1.5 MPa (Zeng et al., 2004). However, *Caragana korshinskii* (Fabaceae)seed germination rate significantly decreases when the water potential is below -0.6 MPa, but approximately 15% seed are still capable of germinating under -2.1 MPa (Zeng et al., 2002). *Cenchrus* sp*inifex* (Gramineae) produces two morphologically distinct types of seeds (large and small). When the soil moisture content is maintained at 20% of the maximum field water-holding capacity, seedlings derived from these two seed types exhibit significant differences in morphological traits (plant height, stem diameter, and root-to-shoot ratio) (Qu and Zhou, 2022).

Our results showed that there were different in the EP and seedling growth of polymorphic seeds under various cultivation conditions. Under moderate NaCl concentration (0.4 mol/L), *Suaeda* sp*lendens* seedlings of polymorphic black and brown seeds grew well; however, seedlings of brown seeds grew less well at lower NaCl concentration (0.2 mol/L) (Redondo-Gómez et al., 2008), indicating that 0.4 mol NaCl/L was a suitable salt concentration. The greenhouse cultivation experiment in the present study showed that under four salt concentration treatments, the EP of YP was significantly higher than that of the other types, with PP having the lowest EP. The main difference was that the SP at low NaCl concentrations (0 and 7 g/kg) were no significant difference. However, at a higher concentration (13 g/kg), YP had the highest EP, but the seedlings could not tolerate the high salt level, resulting in the lowest SP. Thus, in moderate salt conditions, the SP of the polymorphic diaspores did not differ, whereas at higher salt concentrations, YP had the highest germination and emergence ability, but the seedlings had difficulty in surviving.

These differential salt responses of polymorphic seeds have ecological significance in the natural habitat: when the salt condition is suitable, diaspores of YY and YP can emerge and grow well, thereby increasing the seedling survival rate; under salt stress conditions, PP seeds evade salt stress by reducing their germination and emergence ability and form a temporary soil seed bank. When snowmelt/rainfall washes away the surface salt, PP seeds can resume germination and emerge, thereby increasing the survival probability. It has been previously demonstrated that *H. ammodendron* fruits recover germination after salt stress (Zhumabekova et al., 2020).

In the natural habitat of *H. ammodendron*, water stress is prominent. Rapid germination is advantageous when water is available to ensure sufficient seedling growth to withstand subsequent drought stress, whereas slow germination is more advantageous when rainfall is limited (Zeng et al., 2010). The EP of YP was the highest under the three moisture treatments in this study. Under daily watering, YP had most biomass, whereas under watering at 6-day intervals, YY had the highest biomass, indicating it is more drought-resistant.

These differences in the water response of polymorphic seeds also have ecological significance in the natural habitat: in spring, when the soil moisture is sufficient, YP germinates and grows rapidly, thus increasing the seedling numbers and survival probability. However, this also increases the risk when a severe drought occurs subsequently, which would cause mass seedling die-off. When water is insufficient, YY, with better drought tolerance and more robust seedlings, is more likely to survive. Obviously, seed polymorphism in *H. ammodendron* is an effective strategy for adapting to the unpredictable water conditions in the desert. *H. ammodendron* is a Mediterranean relic shrub that has undergone several environmental and geological changes over hundreds of millions of years. Future research perspectives include an in-depth study of what the types of ancestral diaspores were, what event caused them to genetically differentiate, and what are the underlying mechanisms.

## 5 Conclusions

**Figure 5:**
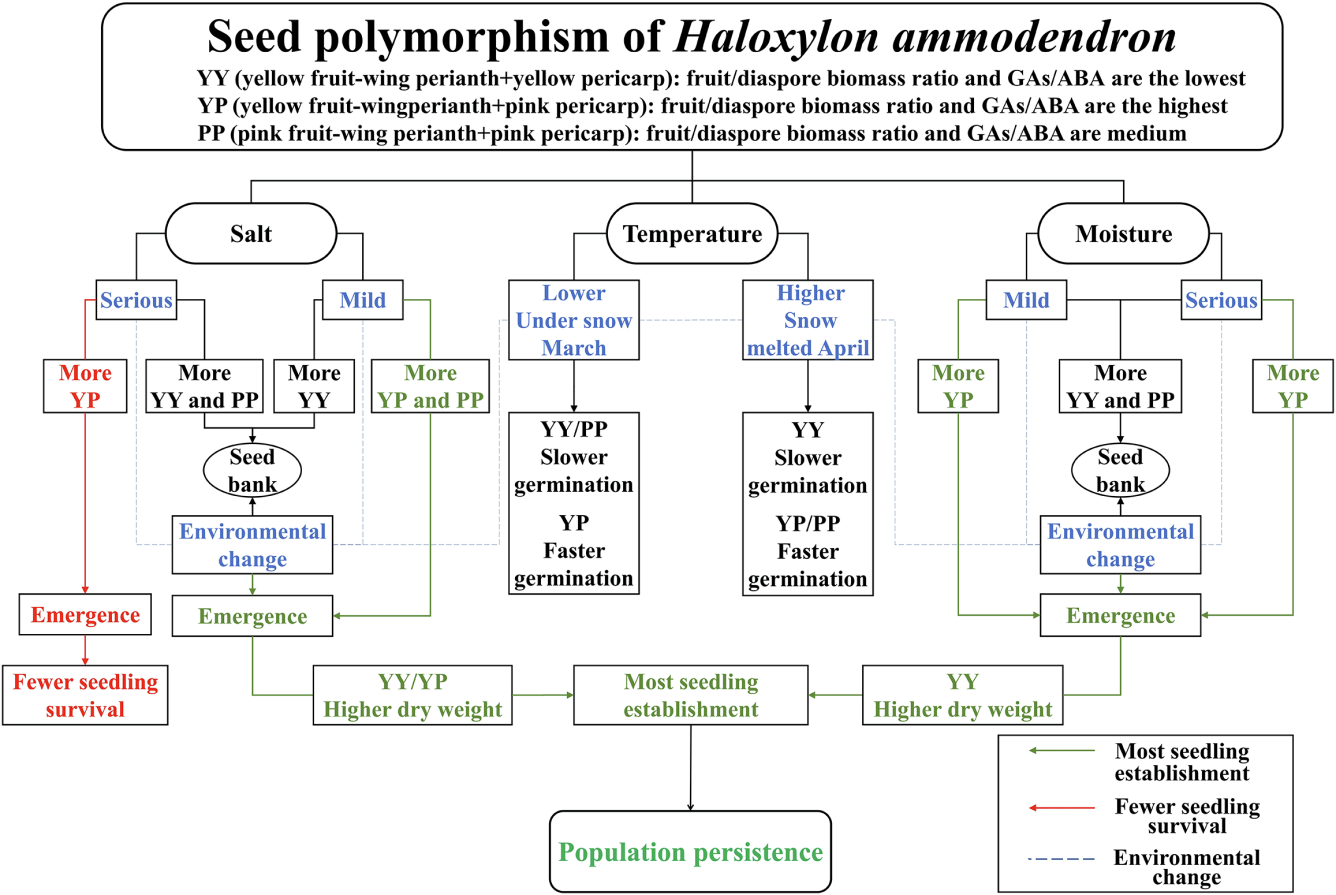
Conceptual model of the dynamics of germination and seedling establishment of polymorphic diaspores of *Haloxylon ammodendron*.

This study revealed that, to adapt to the unpredictable heterogeneous desert environment, the perennial shrub *H. ammodendron* evolved seed polymorphism as a specific bionomic strategy. Its three types of diaspores can effectively respond to environmental changes (**Figure 5**): YY seeds germinate slower, but are more tolerant and robust to ensure a sufficient number of offspring in the harsh environment. YP seeds, with a higher biomass and GA/ABA content ratio, can germinate rapidly under suitable conditions, which contribute to seedling establishment and population expansion. However, their high germination rate results in a waste of seed resources under salt stress, which the seedlings cannot survive. Under high-salt conditions, PP seeds with a low germination rate may form a seed bank to preserve resources and avoid adversity, and resume germination when salt concentrations decrease. This study provides a theoretical basis for studying bionomic strategies to adapt to a harsh environment, as well as germplasm resources (rapid-growth type: YP; stress-resistant type: YY) for the prevention of desertification.

## Data Availability

The raw data supporting the conclusions of this article will be made available by the authors, without undue reservation.
