# Supplementary material for: Haloxylon ammodendron adapts to desert environments through seed polymorphism during diaspore germination and seedling establishment
Source: Front Plant Sci. 2025 Apr 10;16:1527718. doi: 10.3389/fpls.2025.1527718 (PMC12018425; doi:10.3389/fpls.2025.1527718)
Supplement: Supplementary file 1 [file Table1.docx]

Supplementary Material

# Supplementary Figures and Tables

**TABLE S1** 18 investigation populations of *Haloxylon ammodendron* (distributed in Gurbantunggut Desert of the Junggar Basin, Xinjiang, China), population size, polymorhpic plant number and plant ratio. In the investigation point column, abbreviations indicate geographic name: FH for Fuhai, FK for Fukang, FY for Fuyun,WS for Wusu, HTB for Hutubi, JMS for Jimusaer, KLM for Karamay, ML for Mulei, SHZ for Shihezi, QT for Qitai, WJQ for Wujiaqu.

| Investigation point | Longitude (°) | Latitude (°) | Altitude (m) | Total number of plant | YY  (plant Number) | YP  (plant number) | PP  (plant number) | Plant ratio  (YY:YP:PP) |
| --- | --- | --- | --- | --- | --- | --- | --- | --- |
| FH1 | 87.74 | 46.72 | 455.47 | 194 | 47 | 139 | 8 | 1: 3.0: 0.2 |
| FH2 | 87.48 | 47.24 | 452.94 | 362 | 146 | 187 | 29 | 1: 1.3: 0.2 |
| FK1 | 88.13 | 44.31 | 444.97 | 187 | 19 | 123 | 45 | 1: 6.5: 2.4 |
| FK2 | 87.96 | 44.37 | 418.96 | 165 | 31 | 106 | 28 | 1: 3.4: 0.9 |
| FY1 | 88.81 | 46.84 | 674.79 | 117 | 25 | 76 | 16 | 1: 3.0: 0.6 |
| FY1 | 89.51 | 45.82 | 982.92 | 331 | 97 | 186 | 48 | 1: 1.9: 0.5 |
| WS1 | 83.64 | 44.90 | 187.09 | 112 | 36 | 41 | 35 | 1: 1.1: 1.0 |
| HTB1 | 86.89 | 44.67 | 354.12 | 141 | 19 | 66 | 56 | 1: 3.5: 2.9 |
| JMS1 | 86.83 | 47.26 | 585.44 | 123 | 51 | 60 | 12 | 1: 1.2: 0.2 |
| JMS1 | 89.00 | 44.93 | 552.62 | 115 | 9 | 71 | 35 | 1: 7.9: 3.9 |
| KLM1 | 85.33 | 45.52 | 235.52 | 138 | 15 | 113 | 10 | 1: 7.5: 0.7 |
| KLM2 | 85.52 | 45.95 | 300.00 | 248 | 21 | 176 | 51 | 1: 8.4: 2.4 |
| ML1 | 90.90 | 44.40 | 863.21 | 156 | 16 | 99 | 41 | 1: 6.2: 2.6 |
| SHZ1 | 86.31 | 45.01 | 298.99 | 133 | 87 | 35 | 11 | 1: 0.4: 0.1 |
| SHZ2 | 86.14 | 44.72 | 317.57 | 241 | 61 | 131 | 49 | 1: 2.1: 0.8 |
| QT1 | 90.10 | 44.42 | 640.25 | 257 | 35 | 155 | 67 | 1: 4.4: 1.9 |
| WJQ1 | 87.50 | 44.64 | 369.96 | 181 | 37 | 96 | 48 | 1: 2.6: 1.3 |
| WJQ2 | 87.51 | 44.63 | 374.00 | 115 | 32 | 70 | 13 | 1: 2.2: 0.4 |
